# Supplementary material for: An integrated analysis of the structural changes and gene expression of spleen in human visceral leishmaniasis with and without HIV coinfection
Source: PLoS Negl Trop Dis. 2024 Jun 6;18(6):e0011877. doi: 10.1371/journal.pntd.0011877 (PMC11265696; doi:10.1371/journal.pntd.0011877)
Supplement: S1 Table — (PDF) [file pntd.0011877.s001.pdf]

S1_Table. GTEx samples used as healthy liver tissue control.

Tissue Sample ID	Tissue	Subject ID	Sex	Age bracket	Hardy Scale	Pathology Categories	Pathology Notes
GTEX-11LCK-0126	Spleen	GTEX-11LCK	Male	30-39	Ventilator case	no_abnormalities	2 pieces, well-preserved; no abnormalities
GTEX-12WSJ-0526	Spleen	GTEX-12WSJ	Female	40-49	Ventilator case	-	-
GTEX-14DAR-0326	Spleen	GTEX-14DAR	Male	50-59	Ventilator case	no_abnormalities	2 pieces, no abnormalities
