# Supplementary material for: An integrated analysis of the structural changes and gene expression of spleen in human visceral leishmaniasis with and without HIV coinfection
Source: PLoS Negl Trop Dis. 2024 Jun 6;18(6):e0011877. doi: 10.1371/journal.pntd.0011877 (PMC11265696; doi:10.1371/journal.pntd.0011877)
Supplement: S2 Table — (PDF) [file pntd.0011877.s002.pdf]

S2 Table. Housekeeping genes used during normalization

Gene symbol	nCounter ID	Ensembl ID
ABCF1	NM_001090	ENSG00000204574
ALAS1	NM_000688	ENSG00000023330
G6PD	NM_000402	ENSG00000160211
GAPDH	NM_002046	ENSG00000111640
GUSB	NM_000181	ENSG00000169919
HPRT1	NM_000194	ENSG00000165704
OAZ1	NM_004152	ENSG00000104904
POLR1B	NM_019014	ENSG00000125630
POLR2A	NM_000937	ENSG00000181222
PPIA	NM_021130	ENSG00000196262
RPL19	NM_000981	ENSG00000108298
SDHA	NM_004168	ENSG00000073578
TBP	NM_001172085	ENSG00000112592
TUBB	NM_178014	ENSG00000196230
