# Supplementary figures and images for: An integrated analysis of the structural changes and gene expression of spleen in human visceral leishmaniasis with and without HIV coinfection
Source: PLoS Negl Trop Dis. 2024 Jun 6;18(6):e0011877. doi: 10.1371/journal.pntd.0011877 (PMC11265696; doi:10.1371/journal.pntd.0011877)

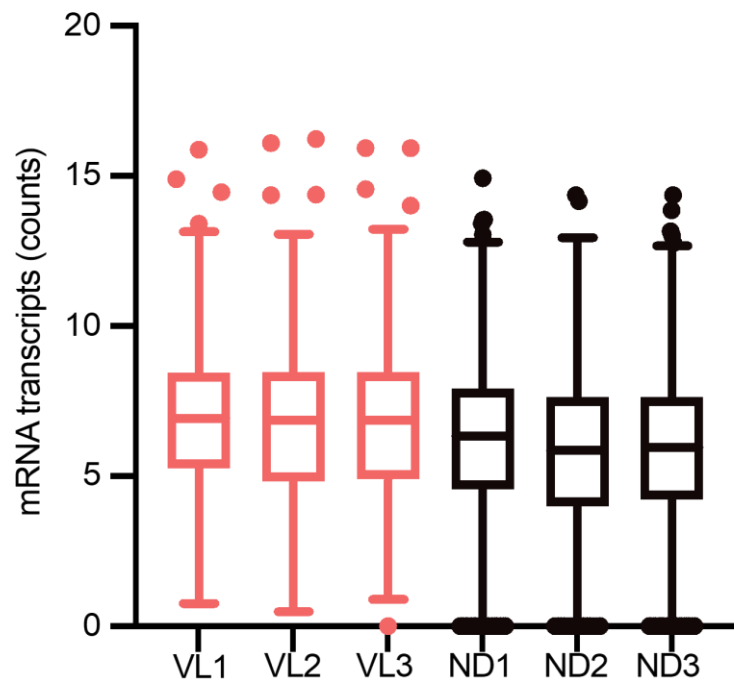

Supplement: S1 Fig — (PDF) [file pntd.0011877.s004.pdf]

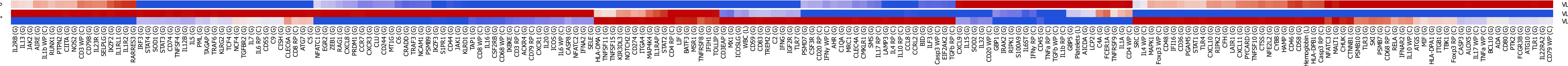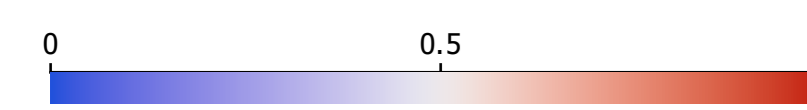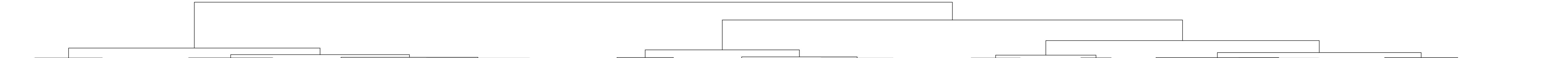

Supplement: S2 Fig — Each row represents each patient (VL1, VL2, VL3), while the number of counts per gene (G), number of cells stained by IHC (C) in WP or RP and laboratorial data (L) are present in columns. The columns were clustered based on Pearson correlation data to each patient. WP- White pulp; RP- Red pulp. (PDF) [file pntd.0011877.s005.pdf]
