# Supplementary material for: An integrated analysis of the structural changes and gene expression of spleen in human visceral leishmaniasis with and without HIV coinfection
Source: PLoS Negl Trop Dis. 2024 Jun 6;18(6):e0011877. doi: 10.1371/journal.pntd.0011877 (PMC11265696; doi:10.1371/journal.pntd.0011877)
Supplement: Attachment — Submitted filename: PNTD-D-23-01629.docx [file pntd.0011877.s006.docx]

The study by Fontes et al., investigates the structural changes and gene expression alterations in the spleens of three patients with relapsing visceral leishmaniasis, two of whom were coinfected with HIV. The IL6 signaling pathway is implicated in disorganizing the white pulp, while BCL10 and ICOSLG are associated with spleen organization. HIV coinfection is linked to lower splenic CD4+ cell density and reduced expression of genes like IL15, potentially compromising the immune response against Leishmania infantum. The study aims to contribute to understanding pathways involved in susceptibility to inadequate responses to L. infantum infection.

While the study provides valuable insights into the structural changes and gene expression alterations in the spleens of patients with relapsing visceral leishmaniasis, there are some potential drawbacks and limitations as discussed below:

Major points:

1. The study is based on a relatively small sample size (which was also acknowledged by the author in the discussion section) of three patients with relapsing visceral leishmaniasis, and only two of them were coinfected with HIV. The limited sample size may affect the generalizability of the findings and limit the statistical power of the analyses.
2. The study could benefit from a more diverse representation of patient characteristics (e.g., Reinaldo et al., 2022), including variations in age, gender, and disease severity. A more heterogeneous sample would enhance the generalizability of the findings to a broader population.
3. The absence of a control group consisting of non-relapsing visceral leishmaniasis cases makes it challenging to delineate specific features associated with relapse. A comparative analysis with non-relapsing cases would provide a more comprehensive understanding of disease dynamics.
4. The clinical correlations in the study, such as treatment responses and disease severity, are mentioned briefly. A more detailed and comprehensive exploration of these clinical parameters and their correlation with gene expression patterns and spleen structure would strengthen the clinical relevance of the study.
5. While the study identifies associations between gene expression changes and clinical outcomes, it falls short of providing detailed mechanistic insights into how these alterations contribute to the pathology of visceral leishmaniasis. Further experiments exploring cellular and molecular mechanisms are warranted. For instance, investigating the specific interactions and signaling pathways involving IL6, BCL10, ICOSLG, and IL15 in the spleen could uncover potential therapeutic targets.
6. Functional studies, such as *in vitro* experiments or animal models, could be conducted to experimentally validate the functional impact of the identified genes on spleen organization and immune response. This would provide a more mechanistic understanding of the observed associations.
7. In Fig 1 the author did not show the staining for CD4 which is an important marker.
