# Supplementary material for: An integrated analysis of the structural changes and gene expression of spleen in human visceral leishmaniasis with and without HIV coinfection
Source: PLoS Negl Trop Dis. 2024 Jun 6;18(6):e0011877. doi: 10.1371/journal.pntd.0011877 (PMC11265696; doi:10.1371/journal.pntd.0011877)
Supplement: Attachment — Submitted filename: Review of PNTD-D-23-01629.docx [file pntd.0011877.s007.docx]

**Review of PNTD-D-23-01629**

**1/30/2024**

Title: An integrated analysis of the structural changes and gene expression of spleen in human visceral leishmaniasis with and without HIV coinfection

Authors: Jonathan L. M. Fontes, Ricardo Khouri, Luis Gustavo C. Reinaldo, Erina M. A. Hassegawa, Antônio José Meneses Filho, Caroline V. B. de Melo, Pablo Ivan P. Ramos, Rafael de Deus Moura, Carla Pagliari, Marta Santos, Raimundo José C. Araújo Jr., Johan Van Weyenbergh, Luiz A. R. de Freitas, Carlos Henrique N. Costa and Washington L. C. dos-Santos

Summary

This is a detailed study of three visceral leishmaniasis (VL) patients, two of whom had HIV co-infection, all three of whom were splenectomized as part of their treatment. Splenectomy permitted extensive histological, cellular and gene expression analysis of the patients. Gene expression in the spleens of three age-matched healthy individuals was used for comparison. In conclusion, the authors present five hypotheses: 1) VL causes spleen disruption 2) disruption is associated with immune exhaustion 3) IL6 plays a role in this process 4) low CD4 count contributes recurrence and 5) impaired IFN-g signaling may lead to Leishmania persistence.

Comments

The continued occurrence of fatal leishmaniasis and the need for a fuller understanding of the disease to aid in the development of prognostic indicators and treatments indicates the importance of studies like this one by Fontes et al. The sample size of three patients, each of which has a slightly different disease state in terms of co-infection with HIV and state of their spleen organization, means the results are only suggestive and would need further confirmation in additional patients. The extensive range of characteristics analyzed including clinical characteristics, histological analysis, immunohistochemistry, gene expression, and an attempt at integrative analysis are the strength of this study. Some weaknesses in the presentation of this data detract from its effectiveness and reduce confidence in the conclusions that are drawn.

Specific Comments

1. In the last paragraph of the Introduction, the authors state, “This study investigates the association between clinical presentation, spleen histology, and gene expression in three patients exhibiting relapsing forms of VL unresponsive to conventional therapy…” The presentation of results highlights the differences among the three patients for clinical characteristics, histology and gene expression patterns, but the associations are not clear in terms of correlation or causation. Furthermore, the “Integrative analysis” which seems to be the approach that would make this analysis more precise, only counts gene expression, not clinical or histological characteristics. The assembly of clusters of genes for each patient is not explained well enough to understand. The dendrogram in Figure S1 is impossible to understand. There is no mention of how Euclidian distance was calculated in the Materials and methods section. Though the count of transcripts per gene is mentioned on line 382, the data is not presented nor a method for using it to produce Figure S1. The legend for Figure S1 suggests the data compiled includes transcripts per gene, number of cells from immunohistochemistry and laboratorial data. If the IHC and laboratorial data is included in the analysis, these features need to be identified in the dendrogram. A reference citation that describes the method should also be added.
2. S1 Figure is presented in the Results section lines 166-179. Lists of genes associated with each patient cluster are presented. However, no clear reason is presented why the particular list is given. For example, the text states patient VL2 is associated with “CXCL9, IL15, SOD2, IL32, GBP1, IRAK2, RIPK1, S100A9, IL6ST, CD45_PTPRC, the cells CD20+ and TGFB+ in WP, cytokines IFNγ, TNFa, and IL1B in RP.” The cluster that represents patient VL2 has 30 genes. The authors should present a reason why the 16 mentioned in the text are selected from among the 30. A similar question could be asked for each of the clusters.
3. In the results presented for the clusters shown in S1 Figure, the authors also mention cells associated with each cluster that were determined by immunohistochemistry. These correlations with patients VL1, VL2 and VL3, are not listed in S1 Figure, but rather in Table 3. This attempt to show a correspondence between the gene expression and the cellular composition of the spleen is an important aspect of this study. However, the presentation of the results must be more clear.
4. Please explain why or at least speculate why no CD4 cells were observed in patient VL2.
5. Line 111 states, “the decreased density observed across most cell populations, including cytokines, FoxP3, and caspase 3-expressing cells, both in the WP and the RP of patient VL1”. The cytokine expressing cells mentioned are definitely reduced as shown in Table 3, however “across most cell populations” is not an accurate statement.
6. Line 117 states ,“CD68+ macrophages were lower” [in VL2]. Is the difference between 151 and 173 significant?
7. Figure 2A is a heatmap of splenic gene expression in the three healthy individuals, and each of the VL patients. There are several questions about this data that should be clarified. 1) The methods do not describe how the RNA samples were prepared. The process of extraction and preparation for the Nanostring assay will affect the measure of transcript per gene. Also, if a piece of spleen tissue is extracted as I expect, the composition of the RNA sample will be an average of the composition of all the cells in the spleen. This will be different from the immunohistochemistry which is able to identify cells individually. This limitation of the comparison should be mentioned. Also to compare the transcripts from healthy spleens in a previous study to transcript in these VL spleen samples, some form of normalization should be performed because the data were acquired by very different means. Describe the normalization performed or justify why it is not needed.
8. In Figure 2A, the heatmap shows approximately 4 bands of color differentiation between each gene name as seen in the screenshot taken from the submitted manuscript. What is the significance of the color changes? Are there genes that are not named? Are there multiple measures of the expression level of each gene? Please clarify.

9. Additionally, VL1 shows a smooth gradation of color change (see screenshot above), not the bands as seen in the other 5 samples. Does this occur because the genes are assembled in the order of expression in VL1? If so mentioning it in the legend would be helpful.
10. Figure 2B shows principal component analysis. There is no mention of PCA in the methods section. At least a citation to the method should be included.
11. Line 156 states that genes upregulated in this study are highlighted in red in figure 2C. This statement is incomplete. Upregulation depends on whether spleen cell were from healthy or VL, and the spleen condition. Please make this statement more accurate.
12. Line 211 states, “IFNG gene appeared highly expressed in VL patients,” S1 Table shows the three VL patients had a higher IFNG than the healthy controls, but there are many other genes in the table with higher expression in VL than control. What is the justification to single out IFNG?
13. Line 214 states, “gene expression pattern in each patient parallels to the number of IFNγ + cells present in the spleen”. This statement does not seem accurate because patient VL3 has the highest number of IFN-g cells, how does the gene expression pattern parallel? VL1 had 2^nd^ highest level of IFN-g cells and lowest IFNG expression (table S1).
14. Line 229 states, “There was an overall increase of CD68+ macrophage compared with the patients with VL and organized spleen.” This statement is missing what the VL patient is compared to.
15. Lines 236-240 state that patient VL1 showed “high density of IL6-producing cells in the RP”. This statement is a correct interpretation of the data (47 cell/mm^2^) in Table 3, however the 37 cell/mm^2^ in white pulp is a larger fold-change compared to the other two patients. The use of the data in the discussion of the study must be consistent and accurate.
16. Line 252 of the Discussion states, “BCL10 and ICOSLG, were highly expressed in type 1 spleen.” This statement is somewhat true based on comparison of the VL patients. However, I assume that the healthy controls had type 1 spleens according to the spleen type rating in Hermida 2018. All healthy controls had these genes expressed at a much lower level. The discussion points must be consistent with the data.
17. Lines 266-270 of the discussion states, “The patient with VL-HIV presented lower density splenic CD4+ cells, low expression of genes such as IL15 and of cytokines related to Th1 response, upregulation of genes related to Th2 response and downregulation of IFNG gene before VL treatment (40) in comparison to the patients with VL monoinfection.” To make this statement consistent with the results, first there were two patients with VL-HIV, which one is this referring to? I assume the statement means lower density of CD4+ cells than the non-HIV-VL patient. If so, the non-HIV patient has higher CD4 in the WP, but lower in the RP. Correct the discussion to be consistent with the results. This quoted sentence also has a citation in the middle making it difficult to understand which points are in reference to the data and which are coming from the literature.
18. Finally, a closing statement that captures the overall significance of the study would improve the paper.

Minor comments

Line 63, the control gene expression profiles are taken from “normal donors”. What is the meaning of “donors” in this sentence?

Line 177, “LV-HIV” should be corrected to VL-HIV

Line 211, states, “hamsters with experimental (29,25)”. Some words seem to be missing. Experimental what?
