# Supplementary material for: An integrated analysis of the structural changes and gene expression of spleen in human visceral leishmaniasis with and without HIV coinfection
Source: PLoS Negl Trop Dis. 2024 Jun 6;18(6):e0011877. doi: 10.1371/journal.pntd.0011877 (PMC11265696; doi:10.1371/journal.pntd.0011877)
Supplement: Attachment — Submitted filename: letter review.docx [file pntd.0011877.s008.docx]

Salvador, March 20th, 2024

To: Dr. Hira L Nakhasi

Section Editor – PLOS Neglected Tropical Diseases

and

Dr. Abhay Satoskar,

Section Editor - PLOS Neglected Tropical Diseases

Dear Dr. Nakhasi and Dr. Satoskar,

I would like to thank you and the reviewers for the detailed revision and important suggestions to the manuscript **“An integrated analysis of the structural changes and gene expression of spleen in human visceral leishmaniasis with and without HIV coinfection”** (PNTD-D-23-01629). Below you can find our detailed responses, highlighted in red, to the reviewers’ comments.

Please feel free to contact me to any further questions.

Sincerely,

Dr. Washington LC dos-Santos

**Answer to Reviewer**

Title: An integrated analysis of the structural changes and gene expression of spleen in human visceral leishmaniasis with and without HIV coinfection

Authors: Jonathan L. M. Fontes, Ricardo Khouri, Luis Gustavo C. Reinaldo, Erina M. A. Hassegawa, Antônio José Meneses Filho, Caroline V. B. de Melo, Pablo Ivan P. Ramos, Rafael de Deus Moura, Carla Pagliari, Marta Santos, Raimundo José C. Araújo Jr., Johan Van Weyenbergh, Luiz A. R. de Freitas, Carlos Henrique N. Costa and Washington L. C. dos-Santos

Summary

This is a detailed study of three visceral leishmaniasis (VL) patients, two of whom had HIV co-infection, all three of whom were splenectomized as part of their treatment. Splenectomy permitted extensive histological, cellular and gene expression analysis of the patients. Gene expression in the spleens of three age-matched healthy individuals was used for comparison. In conclusion, the authors present five hypotheses: 1) VL causes spleen disruption 2) disruption is associated with immune exhaustion 3) IL6 plays a role in this process 4) low CD4 count contributes recurrence and 5) impaired IFN-g signaling may lead to Leishmania persistence.

Comments

The continued occurrence of fatal leishmaniasis and the need for a fuller understanding of the disease to aid in the development of prognostic indicators and treatments indicates the importance of studies like this one by Fontes et al. The sample size of three patients, each of which has a slightly different disease state in terms of co-infection with HIV and state of their spleen organization, means the results are only suggestive and would need further confirmation in additional patients. The extensive range of characteristics analyzed including clinical characteristics, histological analysis, immunohistochemistry, gene expression, and an attempt at integrative analysis are the strength of this study. Some weaknesses in the presentation of this data detract from its effectiveness and reduce confidence in the conclusions that are drawn.

Specific Comments

1. In the last paragraph of the Introduction, the authors state, “This study investigates the association between clinical presentation, spleen histology, and gene expression in three patients exhibiting relapsing forms of VL unresponsive to conventional therapy…” The presentation of results highlights the differences among the three patients for clinical characteristics, histology and gene expression patterns, but the associations are not clear in terms of correlation or causation. Furthermore, the “Integrative analysis” which seems to be the approach that would make this analysis more precise, only counts gene expression, not clinical or histological characteristics. The assembly of clusters of genes for each patient is not explained well enough to understand. The dendrogram in Figure S1 is impossible to understand. There is no mention of how Euclidian distance was calculated in the Materials and methods section. Though the count of transcripts per gene is mentioned on line 382, the data is not presented nor a method for using it to produce Figure S1. The legend for Figure S1 suggests the data compiled includes transcripts per gene, number of cells from immunohistochemistry and laboratorial data. If the IHC and laboratorial data is included in the analysis, these features need to be identified in the dendrogram. A reference citation that describes the method should also be added.

R- The referee is right in her/his comment on the figure S1. Although clinical and morphological data were taken into account in the analysis, they were not properly labeled in the dendrogram. Now we changed the figure to make the information clearer. In this new figure (S2_Fig) the calculations of clustering of columns in heatmap were made by Pearson correlation, note that there is no change in the parameters highlighted by us in the text [Ln. 163-165 and Ln. 617-621].

2. S1 Figure is presented in the Results section lines 166-179. Lists of genes associated with each patient cluster are presented. However, no clear reason is presented why the particular list is given. For example, the text states patient VL2 is associated with “CXCL9, IL15, SOD2, IL32, GBP1, IRAK2, RIPK1, S100A9, IL6ST, CD45_PTPRC, the cells CD20+ and TGFb in WP, cytokines IFNγ, TNFa, and IL1B in RP.” The cluster that represents patient VL2 has 30 genes. The authors should present a reason why the 16 mentioned in the text are selected from among the 30. A similar question could be asked for each of the clusters.

R- In fact, in this section of the text only we presented the data of gene expression that were unique of each patient. Given the small sample and the descriptive character of this study we found relevant to highlight the distinct aspects of gene expression among the patients. Common findings among or between patients are presented in the figure S2. Please, notice that we also highlight the data on the morphology (cells populations and histological organization) and laboratory parameters. In this version of the text we changed the heatmap to better represent the data.

3. In the results presented for the clusters shown in S1 Figure, the authors also mention cells associated with each cluster that were determined by immunohistochemistry. These correlations with patients VL1, VL2 and VL3, are not listed in S1 Figure, but rather in Table 3. This attempt to show a correspondence between the gene expression and the cellular composition of the spleen is an important aspect of this study. However, the presentation of the results must be more clear.

R- We agree with the referee. Now we changed the figure to better represent the relevant results. All the relevant information is now in the caption of the figure [Ln. 617-621].

4. Please explain why or at least speculate why no CD4 cells were observed in patient VL2.

R- In fact CD4 cells were present in all the patients. However, this information may have not been properly shown. Now we change the Fig 1. and added a phrase in the text to emphasize this information [Ln. 102 and Ln. 114].

5. Line 111 states, “the decreased density observed across most cell populations, including cytokines, FoxP3, and caspase 3-expressing cells, both in the WP and the RP of patient VL1”. The cytokine expressing cells mentioned are definitely reduced as shown in Table 3, however “across most cell populations” is not an accurate statement.

R- We agree, and changed to “across some leukocytes population” [Ln. 111].

6. Line 117 states ,“CD68+ macrophages were lower” [in VL2]. Is the difference between 151 and 173 significant?

R – In fact, these number are only descriptive of two patients. Therefore, statistical compression is not possible. We softened the phrase in to better express the uncertainty of a descriptive data based in such a small sample.

7. Figure 2A is a heatmap of splenic gene expression in the three healthy individuals, and each of the VL patients. There are several questions about this data that should be clarified. 1) The methods do not describe how the RNA samples were prepared. The process of extraction and preparation for the Nanostring assay will affect the measure of transcript per gene. Also, if a piece of spleen tissue is extracted as I expect, the composition of the RNA sample will be an average of the composition of all the cells in the spleen. This will be different from the immunohistochemistry which is able to identify cells individually. This limitation of the comparison should be mentioned. Also to compare the transcripts from healthy spleens in a previous study to transcript in these VL spleen samples, some form of normalization should be performed because the data were acquired by very different means. Describe the normalization performed or justify why it is not needed.

R- We appreciate the opportunity to clarify these points and enhance the quality of our work. You are right in highlighting the resolution advantage that single-cell immunohistochemistry provides over bulk RNA sequencing techniques. Immunohistochemistry allows for the analysis of gene expression at the individual cell level, offering a more granular view of cellular heterogeneity within tissues. This is particularly relevant in complex tissues like the spleen, where the bulk RNA-seq approach might mask the contributions of distinct cell types to the overall gene expression profile. We concur with your assessment and have included a discussion in the revised manuscript to address this important comparison. Regarding the RNA sample preparation and normalization procedures, we acknowledge that these details were initially omitted from the methods section. To rectify this, we have now included a comprehensive description of the RNA extraction and preparation process for the Nanostring assay in the revised manuscript. As you rightly pointed out, the nature of the sample—being derived from a heterogeneous tissue—necessitates a careful approach to ensure that the RNA profile accurately reflects the composition of the spleen. Furthermore, we have addressed the issue of normalization, which is crucial for making meaningful comparisons between the transcriptomes of healthy spleens from previous studies and those affected by visceral leishmaniasis (VL) in our current study. We have implemented a normalization procedure to account for the differences in assay techniques and sample preparations, ensuring that the comparisons are valid and informative. The details of this normalization process have been added to the methods section of our manuscript. Also, as we updated the statistical calculations, we have noted a slight change in the data, where three genes were not differentially expressed anymore (171 DEGs, now 168 DEGs). However, this change did not compromise the principal message of this manuscript. [Ln. 136 and Ln. 386-434]

8. In Figure 2A, the heatmap shows approximately 4 bands of color differentiation between each gene name as seen in the screenshot taken from the submitted manuscript. What is the significance of the color changes? Are there genes that are not named? Are there multiple measures of the expression level of each gene? Please clarify.

R- Each band correspond to a different gene. We have change the heatmap to make possible to name each one of them in the figure. However, a complete DEGs list with their log2FC is provided in S3_Table. Now we added an explanatory phrase in the caption of figure2A including this information [Ln. 148].

9. Additionally, VL1 shows a smooth gradation of color change (see screenshot above), not the bands as seen in the other 5 samples. Does this occur because the genes are assembled in the order of expression in VL1? If so mentioning it in the legend would be helpful.

R- The genes were assembled in the order of expression in patient VL1. The suggestion of the referee was accepted [Ln. 148-150].

10. Figure 2B shows principal component analysis. There is no mention of PCA in the methods section. At least a citation to the method should be included.

R- This is now mentioned in the methodology [Ln. 432-435].

11. Line 156 states that genes upregulated in this study are highlighted in red in figure 2C. This statement is incomplete. Upregulation depends on whether spleen cell were from healthy or VL, and the spleen condition. Please make this statement more accurate.

R- This has been done [Ln. 157].

12. Line 211 states, “IFNG gene appeared highly expressed in VL patients,” S1 Table shows the three VL patients had a higher IFNG than the healthy controls, but there are many other genes in the table with higher expression in VL than control. What is the justification to single out IFNG?

R- The referee is right. Many other genes had higher expression in the spleens of patients with VL than in the controls. However, we only highlighted *IFNG* because only for this gene we also had the immunohistochemistry staining confirming the data. We included an explanatory note in the text [Ln. 214].

13. Line 214 states, “gene expression pattern in each patient parallels to the number of IFNγ + cells present in the spleen”. This statement does not seem accurate because patient VL3 has the highest number of IFN-g cells, how does the gene expression pattern parallel? VL1 had 2^nd^ highest level of IFN-g cells and lowest IFNG expression (table S1).

R- The referee is right. Only in patient VL3 the *IFNG* expression parallels to the number of IFNg-expressing cells in immunohistochemistry. This statement has been corrected in the discussion [Ln. 218].

14. Line 229 states, “There was an overall increase of CD68+ macrophage compared with the patients with VL and organized spleen.” This statement is missing what the VL patient is compared to.

R- This comment relates the patient VL1 with the other VL patients. The statement is now corrected in the text [Ln. 233].

15. Lines 236-240 state that patient VL1 showed “high density of IL6-producing cells in the RP”. This statement is a correct interpretation of the data (47 cell/mm^2^) in Table 3, however the 37 cell/mm^2^ in white pulp is a larger fold-change compared to the other two patients. The use of the data in the discussion of the study must be consistent and accurate.

R- The referee is right. The correct information is “high density of IL6-producing cells in the RP and WP.” This has been corrected in the text [Ln. 243].

16. Line 252 of the Discussion states, “BCL10 and ICOSLG, were highly expressed in type 1 spleen.” This statement is somewhat true based on comparison of the VL patients. However, I assume that the healthy controls had type 1 spleens according to the spleen type rating in Hermida 2018. All healthy controls had these genes expressed at a much lower level. The discussion points must be consistent with the data.

R- In fact BCL10 and ICOSLG genes are associated with secondary lymphoid follicles. Type 1 spleen contains secondary lymphoid follicles, sometimes highly hyperplastic. This finding is not always present in health individuals. In some health individuals we have found only primary or small reactive follicles. We consider that the spleens of our VL patients may represent different stages of changes produced by VL in the spleen (hyperplasia an atrophy and disorganization). The text was changed to make the comment clear [Ln. 261-262].

17. Lines 266-270 of the discussion states, “The patient with VL-HIV presented lower density splenic CD4+ cells, low expression of genes such as IL15 and of cytokines related to Th1 response, upregulation of genes related to Th2 response and downregulation of IFNG gene before VL treatment (40) in comparison to the patients with VL monoinfection.” To make this statement consistent with the results, first there were two patients with VL-HIV, which one is this referring to? I assume the statement means lower density of CD4+ cells than the non-HIV-VL patient. If so, the non-HIV patient has higher CD4 in the WP, but lower in the RP. Correct the discussion to be consistent with the results. This quoted sentence also has a citation in the middle making it difficult to understand which points are in reference to the data and which are coming from the literature.

R – These corrections have been made [Ln. 270-275].

18. Finally, a closing statement that captures the overall significance of the study would improve the paper.

R- A phrase was added that summarize the contribution of the study [Ln. 282-289].

Minor comments

Line 63, the control gene expression profiles are taken from “normal donors”. What is the meaning of “donors” in this sentence?

R – We included in the method how we obtained the data from healthy patients [Ln. 411-419].

Line 177, “LV-HIV” should be corrected to VL-HIV

R – This has been done [Ln. 178].

Line 211, states, “hamsters with experimental (29,25)”. Some words seem to be missing. Experimental what?

R – The information has been completed in the text [Ln. 214].

**Answer to Reviewer**

The study by Fontes et al., investigates the structural changes and gene expression alterations in the spleens of three patients with relapsing visceral leishmaniasis, two of whom were coinfected with HIV. The IL6 signaling pathway is implicated in disorganizing the white pulp, while BCL10 and ICOSLG are associated with spleen organization. HIV coinfection is linked to lower splenic CD4+ cell density and reduced expression of genes like IL15, potentially compromising the immune response against Leishmania infantum. The study aims to contribute to understanding pathways involved in susceptibility to inadequate responses to L. infantum infection.

While the study provides valuable insights into the structural changes and gene expression alterations in the spleens of patients with relapsing visceral leishmaniasis, there are some potential drawbacks and limitations as discussed below:

Major points:

1. The study is based on a relatively small sample size (which was also acknowledged by the author in the discussion section) of three patients with relapsing visceral leishmaniasis, and only two of them were coinfected with HIV. The limited sample size may affect the generalizability of the findings and limit the statistical power of the analyses.

R- The referee is right. As we mention in the manuscript, the sample is small, and statistics is not possible. However, the paper adds information to the qualitative changes already known to be present in VL and reinterpret these data using new techniques. For this reason, even considering this a small sample, we found worth to submit this new information to analysis of the scientific community.

2. The study could benefit from a more diverse representation of patient characteristics (e.g., Reinaldo et al., 2022), including variations in age, gender, and disease severity. A more heterogeneous sample would enhance the generalizability of the findings to a broader population.

R- We thank the referee for this comment. In fact, a work is now underway between our group and the group lead by Prof. Carlos Henrique Nery Costa using a larger sample, that includes some of the patients whose data was published by Reinaldo et al 2022.

3. The absence of a control group consisting of non-relapsing visceral leishmaniasis cases makes it challenging to delineate specific features associated with relapse. A comparative analysis with non-relapsing cases would provide a more comprehensive understanding of disease dynamics.

R- The referee is right. At this stage it is not possible to delineate specific features associated with VL relapse. It also will be difficult to obtain (here in Brazil) samples from patients that cure from the disease. However, recently we obtained spleen samples of 18 patients that died of VL. These samples are formalin fixed, paraffin embedded tissue. We expect that the study of this samples together with a comparative analysis of spleen removed due trauma may help to understand aspects of the different forms of VL.

4. The clinical correlations in the study, such as treatment responses and disease severity, are mentioned briefly. A more detailed and comprehensive exploration of these clinical parameters and their correlation with gene expression patterns and spleen structure would strengthen the clinical relevance of the study.

R- A comment integrating clinical and gene expression was added to the discussion [Ln. 281-288]

5. While the study identifies associations between gene expression changes and clinical outcomes, it falls short of providing detailed mechanistic insights into how these alterations contribute to the pathology of visceral leishmaniasis. Further experiments exploring cellular and molecular mechanisms are warranted. For instance, investigating the specific interactions and signaling pathways involving IL6, BCL10, ICOSLG, and IL15 in the spleen could uncover potential therapeutic targets. Functional studies, such as *in vitro* experiments or animal models, could be conducted to experimentally validate the functional impact of the identified genes on spleen organization and immune response. This would provide a more mechanistic understanding of the observed associations.

R- We agree with the referee about the relevance of these molecules in the process of changes that occur in the spleen in infectious disease, including VL. The significance of pathways involving IL6 and B-cell differentiation pathways have emerged in other studies by our and other groups, performed in human or experimental animal specimens [ <https://doi.org/10.1084/jem.20170580>, <https://doi.org/10.1111/pim.12455> ]. Our group has been exploring these spleen changes associated with VL in experiments with mice and hamsters. Some results of these studies are under analysis. Partial results have been already published (please see <https://doi.org/10.3389/fimmu.2021.716314> and <https://doi.org/10.3389/fimmu.2020.00653>). A phrase highlighting this aspect was added to the discussion [Ln. 208-212].

7. In Fig 1 the author did not show the staining for CD4 which is an important marker.

R – This is now presented in the revised version of the manuscript [Ln. 102]

We thank the referee for their detailed analysis of our manuscript.
